# Side-by-Side Comparison of Culture Media Uncovers Phenotypic and Functional Differences in Primary Mouse Aortic Mural Cells

**DOI:** 10.3390/cells14120927

**Published:** 2025-06-19

**Authors:** Iman Ghasemi, Rajinikanth Gogiraju, Sana’a Khraisat, Sven Pagel, Claudine Graf, Moritz Brandt, Thati Madhusudhan, Philip Wenzel, Guillermo Luxán, Philipp Lurz, Magdalena L. Bochenek, Katrin Schäfer

**Affiliations:** 1 Department of Cardiology, Cardiology I, University Medical Center of the Johannes Gutenberg University, D-55131 Mainz, Germany; iman.ghasemi@unimedizin-mainz.de (I.G.); rajinikanth.gogiraju@unimedizin-mainz.de (R.G.); moritz.brandt@unimedizin-mainz.de (M.B.); wenzelp@uni-mainz.de (P.W.); lurzphil@uni-mainz.de (P.L.); 2 Center for Thrombosis and Hemostasis, University Medical Center of the Johannes Gutenberg University, D-55131 Mainz, Germany; sanaa.khraisat@unimedizin-mainz.de (S.K.); sven.pagel@unimedizin-mainz.de (S.P.); claudine.graf@unimedizin-mainz.de (C.G.); m.thati@uni-mainz.de (T.M.); magdalena.bochenek@unimedizin-mainz.de (M.L.B.); 3 Institute of Cardiovascular Regeneration, Center of Molecular Medicine, Goethe University Frankfurt, D-60629 Frankfurt, Germany; luxan@med.uni-frankfurt.de; 4 Cardiopulmonary Institute, Goethe University Frankfurt, D-60629 Frankfurt, Germany

**Keywords:** smooth muscle cells, fibroblasts, pericytes, phenotypic plasticity, vascular remodeling, proliferation, migration, metabolism

## Abstract

(1) Background: Vascular mural cells reside in the media and outer layers of the vessel wall. Their ability to proliferate and migrate or to change phenotype in response to external cues is a central feature of the vascular response to injury. Genetically engineered mice are used for loss- or gain-of-function analyses or lineage tracing in vivo, their primary cells for mechanistic studies in vitro. Whether and how cultivation conditions affect their phenotype and function is often overlooked. (2) Methods: Here, we systematically studied how the cultivation of primary mural cells isolated from the aorta of adult wild-type mice in either basal medium (DMEM) or special media formulated for the cultivation of fibroblasts or pericytes affects their phenotype and function. (3) Results: Medium composition did not alter cell viability, but the mRNA levels of differentiated smooth muscle cell markers were highest in vascular mural cells expanded in DMEM. Conversely, significantly higher numbers of proliferating and migrating cells were observed in cells expanded in Pericyte medium, and cytoskeletal rearrangements supported increased migratory capacities. Significantly reduced telomere lengths and metabolic reprogramming was observed in aortic mural cells cultured in Fibroblast medium. (4) Conclusions: Our findings underline the plasticity of primary aortic mural cells and highlight the importance of the culture media composition during their expansion, which could be exploited to interrogate their responsiveness to external stimuli or conditions observed in vivo or in patients.

## 1. Introduction

Vascular mural cells reside in the vessel wall, with smooth muscle cells (SMCs) primarily in the media of large arteries, like the aorta, and fibroblasts or pericytes predominantly in the adventitia [1]. Specific subpopulations of SMCs are also found in the adventitia [2]. Together with endothelial cells, these vascular mural cell types maintain the structural integrity of the vessel wall and vascular homeostasis [3].

Following vascular injury or damage, vascular mural cells respond by adapting their differentiation and activation state to enable proliferation, migration, and other processes to initiate a vascular healing response. This cellular adaptation to external stimuli or damage cues is known as ‘phenotype switching’ and has been best described for vascular SMCs [4,5]. While this process is central for vascular repair and regeneration, dysregulation or overactivation can tilt the balance toward vascular damage or degeneration. In fact, the plasticity of vascular SMCs and their ability to change from a differentiated, contractile, and quiescent state toward a proliferative, synthetic and activated state plays an important role in several cardiovascular disease pathologies, including atherosclerosis and restenosis [6,7,8].

Vascular SMCs respond to cytokines or growth factors released from inflammatory cells or activated platelets, like platelet-derived growth factor (PDGF) or fibroblast growth factor (FGF), with a more proliferative and migratory behavior and an increase in the expression of extracellular matrix (ECM) proteins. The secretion of ECM components is also the main function of fibroblasts, the principal cell type within connective tissue, providing mechanical support to vascular structures. Vascular injury also leads to fibroblast activation and the transition into myofibroblasts [9]. While an important part of the vascular healing response, SMC and fibroblast activation and changes in ECM composition may increase vascular stiffness [10] or alter the activity and recruitment of immune cells [11,12,13], whereas mural cell loss may result in aneurysm formation [14,15].

Primary cells isolated from the arterial tissues of wild-type or transgenic mice are frequently used as a model system to study specific molecular mechanisms in cells or the effects of an experimental intervention on cardiovascular and other disease processes. Optimal medium conditions for the cultivation and expansion of primary arterial mural cells were described more than forty years ago [16], laying the basis for current daily laboratory practice. However, our understanding of vascular cells and (cardiovascular) disease mechanisms as well as potential therapeutic strategies and approaches to target them has increased since then, and technical advances and the availability of novel experimental readouts make a re-evaluation and comparison of different cell culture media timely and necessary.

Here, we systematically examined and compared the phenotype and function of primary vascular mural cells isolated from the aortas of adult wild-type mice in response to three different cell culture media, focusing on key cellular features, such as viability, proliferation, apoptosis, senescence, migration, differentiation, and metabolism. Dulbecco’s Modified Eagle Medium (DMEM) supplemented with 10% fetal bovine serum, supporting the growth of many different mammalian cells, was compared to specialized cell culture media optimized for the expansion of fibroblasts or pericytes, respectively. Our findings that medium composition impacts the proliferation, migration, metabolism, and differentiation state of primary aortic mural cells support their continuing plasticity and ability to respond to culture medium conditions, which is not only relevant for interpreting experimental findings, but may also be exploited to examine their response to external stimuli or damage cues observed in animal models or in patients in vitro.

## 2. Materials and Methods

### 2.1. Primary Aortic Mural Cell Isolation and Cultivation

Male C57/BL6 mice (Janvier Labs; in-house breeding; *n* = 19 biological replicates in total, examined in batches of three to four mice per isolation; mean age: 16.3 ± 0.73 weeks; mean body weight: 28.7 ± 0.97 g) were killed by cervical dislocation under deep inhalation anesthesia (100% isoflurane; Cat. No. 9714675; Piramal Critical Care Deutschland GmbH, Hallbergmoos; Germany). Mice were housed in type 2 polycarbonate cages lined with Aspen chips and equipped with a food hopper and water bottle and containing tunnels and additional bedding materials as environmental enrichment. Mice had free, unlimited access to food and water. The temperature was constantly set to 22 °C, and lights were set to maintain a 12 h light (from 6:00 a.m. to 6:00 pm.) -dark cycle. Mice were fed a standard grain-based laboratory diet (V1124-300; Ssniff Speizialdiäten GmbH, Soest, Germany). Then the abdominal and the thoracic cavity were opened via a longitudinal incision, the inferior vena cava was cut to interrupt the venous flow back to the heart, and the mice were slowly perfused with 2 mL NaCl (B. Braun, D-34212 Melsungen, Germany; Cat. No. 347873) using a 20 G needle (B. Braun, Cat. No. 4657519) inserted into the left ventricle. Next, the entire aorta (excluding the arch) was carefully dissected and cleaned from perivascular adipose tissue and minced using fine scissors (Fine Science Tools, D-69115 Heidelberg, Germany; Cat. No. 14028-10). Pieces of aortic tissue were immediately subjected to enzymatic digestion in collagenase type II (Gibco via Thermo Fisher Scientific, Waltham, MA 02451, USA; Cat. No. 17101015; 1 mg/mL in Dulbecco’s Modified Eagle Medium [DMEM] plus 10% fetal bovine serum [FBS; Gibco via Thermo Fisher Scientific, Waltham, MA 02451, USA; Cat. No. A5256701]) for 3 h at 37 °C with constant shaking (300 rpm). Cell homogenates were centrifuged at 1500× *g* at room temperature (RT) for 5 min to separate cells (pellet) from non-cellular material, such as protein and lipids (supernatant). The supernatant was discarded and the pellet was suspended in 1 mL DMEM, divided into three portions of 300 µL in 2 mL tubes, centrifuged again (1500× *g*, 5 min), and finally randomly resuspended in 1 mL of either DMEM (Gibco via Thermo Fisher Scientific, Waltham, MA 02451, USA, Cat. No. 31966021) supplemented with 10% of heat inactivated (60 °C for 30 min) FBS or in one of the two special culture media for the cultivation and expansion of vascular mural cells: Fibroblast Growth Medium 2 (PromoCell, D-69129 Heidelberg, Germany; Cat. No. C-23120), containing 2.5% FCS (PromoCell, D-69129 Heidelberg, Germany; Cat. No. C-37320), basic fibroblast growth factor (FGF; 1 ng/mL; PromoCell, D-69129 Heidelberg, Germany; Cat. No. C-30310) and insulin (5 μg/mL; PromoCell, D-69129 Heidelberg, Germany; Cat. No. C-3110), or Pericyte medium (ScienCell, Carlsbad, CA 92008, USA; Cat. No. 1201) consisting of basal medium, 2% FBS (ScienCell, Carlsbad, CA 92008, USA; Cat. No. 0010) and 1% pericyte growth supplement (ScienCell, Carlsbad, CA 92008, USA; Cat. No. 1252). All culture media contained 1% penicillin/streptomycin (Gibco via Thermo Fisher Scientific, Waltham, MA 02451, USA; Cat. No. 15140122). Depending on the planned experiments, cells were seeded in the appropriate densities on plastic plates (with 96, 12, or 6 wells per plate) or on 13 mm glass slides coated with 0.2% gelatin (Sigma-Aldrich, D-82024 Taufkirschen, Germany; Cat. No. G1393). The cells were incubated at 37 °C with 5% CO_2_ concentration. Cells were inspected daily to observe their shapes and confluencies in an unblinded manner. Fresh medium was provided every two days to replace nutrients and to maintain optimal pH conditions. The cells were not passaged (except for the MTS and the Seahorse assay, for which the cells needed to be counted and re-plated at equal densities) and analyzed in parallel between days 4 and 6 after isolation after having reached the appropriate density (70–80% for proliferation assays, 95–100% for migration assays, and 80–90% for all others). Experiments were carried out using primary aortic cells obtained from distinct mice (biological replicates) and repeated per batch of mice, with consistent results across experimental repeats. To reduce potential bias, the quantitative analysis of the experimental readouts was conducted by a person who did not participate in the cell preparation and cultivation. All experiments involving animals had been á priori approved by the Translational Animal Research Committee of the University of Mainz and the Landesuntersuchungsamt Rheinland-Pfalz (animal permit G22-1-032) and complied with national guidelines for the care and use of laboratory animals. All in vivo experiments were reported according to the ARRIVE guidelines [17].

### 2.2. Analysis of Cell Viability

Cell viability was examined using the MTS assay (Promega, GmbH, D-69190 Walldorf, Germany; Cat. No. C3582). A total of 1 × 10^5^ cells in 100 µL medium were cultivated for 48 h at 37 °C/5% CO_2_ on gelatin-coated 96-well plates (Thermo Scientific, Waltham, MA 02451, USA; Cat. No. 167008). Then, 20 µL of MTS reagent was added and the cells were incubated for an additional 4 h. The formazan product was measured using the SpectraMax MiniMax 300 Imaging Cytometer (Molecular Devices, Sunnyvale, CA 94085, USA) at 490 nm wavelength. The number of total, viable, and dead cells was also automatically quantified using the Spark^®^ multimode microplate reader (Tecan Life Science, D-74564 Crailsheim, Germany; Cat. No. 30124652). For this, cells were trypsinized (using 0.05% trypsin [Gibco via Thermo Fisher Scientific, Waltham, MA 02451, USA; Cat. No. 25300054]; 600 µL per well of a 6-well plate) for 15 min at 37 °C and mixed in specific medium. Then, 10 μL of the mixed cells was added to 10 μL of Trypan Blue solution (Sigma-Aldrich, D-82024 Taufkirschen, Germany, Cat. No. 72-57-1) and loaded onto a Dual chamber cell chip (Tecan Life Science, D-74564 Crailsheim, Germany; Cat. No. TE18811). The data were analyzed using SPARKCONTROL software (version 2.3)(Tecan Life Science, D-74564 Crailsheim, Germany).

### 2.3. Detection of Apoptotic Cells

Apoptotic cells were detected using the Terminal deoxynucleotidyl transferase dUTP nick-end labeling (TUNEL) method (In Situ Cell Death Detection Kit; Roche, Cat. No. 11684795910) following the manufacturer’s instructions. In brief, cells were fixed with 2% paraformaldehyde (PFA; Sigma-Aldrich, D-82024 Taufkirschen, Germany, Cat. No. 158127) for 20 min at RT, permeabilized with 0.1% Triton X-100 (Carl Roth GmbH, D76185 Karlsruhe, Germany, Cat. No. 3051.3), and incubated with the TUNEL reaction mixture for 1 h. Then the samples were washed once in phosphate-buffered saline (PBS; ThermoFisher Scientific, Waltham, MA, USA; Cat. No. 14190094) and counterstained with 4′,6-diamidino-2-phenylindole (DAPI; Carl Roth GmbH, Karlsruhe, Germany; Cat. No. 63351; dilution, 1:1000) before being analyzed under a fluorescence microscope. The results were quantified by manually counting the number of TUNEL-positive cell nuclei per total cell nuclei per microscope field at 20× magnification.

### 2.4. Detection of Senescent Cells

Senescence-associated beta-galactosidase (SA-β-gal) activity, measurable at pH 6.0, was used to identify senescent cells in culture. For this, cells were seeded onto 0.2% gelatin-coated 12-well plates (VWR International GmbH, D-64295 Darmstadt, Germany; Cat. No. 734-2325), cultured for 5 days, and fixed using the fixation buffer supplied with the senescence detection kit (abcam, CD4 OFW Cambridge, UK; Cat. No. ab65351). Following an overnight incubation with the staining solution at 37 °C in a non-CO_2_ incubator, senescent cells were detected using an inverted brightfield microscope (Motic AE31; Motic Europe GmbH, D35578 Wetzlar, Germany). The number of blue, SA-β-gal positive, senescent cells was manually counted and quantified as the percentage of total cells per microscope field at 10× magnification.

### 2.5. Fluorescence in Situ Hybridization Analysis of Telomere Length

Fluorescence in situ hybridization (FISH) with peptide nucleic acid probes against the C-rich strand of telomeres (5′CCCTAA′3 repeats; TelC-Cy3; PNA bio, Thousand Oaks, CA 91320. USA; Cat. No. F1002) was employed to determine the telomere length following the manufacturer’s instructions. Briefly, cells were grown on glass coverslips to 80% confluency, washed three times with PBS, and fixed with MeOH:EtOH (1:1 vol/vol) for 20 min at −20 °C, followed by three washes with PBS. The cells were rehydrated with a series of decreasing ethanol concentrations (100%, 85%, 70%, 50%, and 30% ethanol, 2 min each) and washed in PBS. Then, cells were incubated with RNase solution (RNase A, 10 mg/mL; Thermo Fisher Scientific, Waltham, MA, USA; dilution, 1:100 in PBS) for 10 min at 37 °C, washed twice with PBS, serially dehydrated using 70%, 85%, and 100% cold EtOH for 2 min each, and air dried. Then, coverslips (with cells) were pre-warmed at 80 °C for 5 min, 15 µL of hybridization buffer (20 mM Tris, pH 7.4, 60% formamide, 0.5% blocking reagent [Roche Diagnostics GmbH, D-68305 Mannheim, Germany; Cat. No. 11096176001]) containing 500 nM TelC-Cy3 probe were added to each coverslip and incubated in a humidified chamber at 80 °C for 10 min, then at RT for 2 h in the dark. The coverslips were washed in wash solution (2× SSC, 0.1% Tween-20) twice for 10 min each at 60 °C, and one last wash at RT. Cells were counterstained with DAPI (Thermo Fisher Scientific, Waltham, MA, USA; Cat. No. D1306; dilution, 1:1000 in 2× SSC) for 15 min, then washed with 2× SSC, 1× SSC, and water for 2 min each. Mounting media (Dako, DK-2600 Glostrup, Denmark; Cat. No. S302380-2) was added and coverslips were mounted cell-side down on glass slides. The next day, the slides were imaged using fluorescence microscopy (Keyence, D-68263 Neu-Isenburg, Germany; BZ-X810). The total telomere signal of 200 nuclei was quantified per sample and condition by measuring the total fluorescence intensity in each image, calibrated for the DAPI signal, using ImageJ (version 1.54f).

### 2.6. Immunofluorescence Staining of Proliferation and Mural Cell Lineage Markers

For immunofluorescence stainings, cells were cultivated in 6-well plates (ThermoFisher Scientific, Waltham, MA, USA; Cat. No. 657160) containing three 0.2% gelatin-coated glass coverslips per well. At 80–90% confluency (70–80% for Ki-67 staining), cells were carefully washed with PBS and fixed using 4% PFA for 15 min at RT. To facilitate antibody penetration, cells were permeabilized using a 0.1% Triton X-100 for 15 min at RT. Cells were incubated with primary antibodies against Ki-67 (abcam, Cambridge, UK; ab15580; dilution, 1:100), NG2 (abcam, Cambridge, UK; ab129051; dilution, 1:100), PDGFRA (Pierce via Thermo Fisher Scientific, Rockford, IL, USA; PA516742; dilution, 1:100), or SMA (Sigma-Aldrich, D-82024 Taufkirschen, Germany, A2547; dilution, 1:100). Unbound primary antibodies were removed by washing with PBS four times. Next, fluorescence-labeled secondary antibodies were added (Invitrogen, ThermoFisher Scientific, Waltham, MA, USA; A-5090 Lofer, Austria; Alexa Fluor 555, Cat. No. A32732; dilution, 1:300). DAPI was added together with the secondary antibody solution to simultaneously stain DNA in cell nuclei. Coverslips with stained cells were inverted and mounted onto glass slides in fluorescent mounting medium (Dako, Cat. No. S3023). Images were taken on an inverted fluorescence microscope (Keyence, D-68263 Neu-Isenburg, Germany; model BZ-X810).

### 2.7. Image Quantification of Cellular Fluorescence

The cellular expression of a particular antigen of interest (as detected by fluorescent probe binding [e.g., fluorescein phalloidin] or immunostaining with fluorescence-labeled antibodies) was quantified by measuring the area and the integrated density using ImageJ software. After capturing the images using an inverted fluorescence microscope (Keyence, D-68263 Neu-Isenburg, Germany; model BZ-X810), immunopositive cells or the total image area were selected using the drawing tool from the menu, and the area and the integrated intensity were automatically measured. Cells or regions without signal were measured as background signal and subtracted from the selected area of fluorescence using this formula: Integrated Density—marked background area. Data are expressed as ‘Fluorescence Integrated Density’ (IntDen).

### 2.8. Flow Cytometry Analysis

To harvest cells from the culture dish, cells were incubated with 0.05% trypsin (600 µL per well of a 6-well plate) for 20 min at 37 °C. To check their viability, cells were incubated with a viability dye (Fixable Viability Dye eFluor™780; eBioscience™ via ThermoFisher Scientific, Waltham, MA, USA) for 30 min at RT under exclusion of light. Unspecific binding was blocked using CD16/CD32 blocking reagent (CD16/CD32 Monoclonal Antibody, eBioscience™, San Diego, CA, USA), followed by incubation with primary antibody against PDGFRA (Pierce via Thermo Fisher Scientific, Rockford, IL, USA; PA516742; dilution, 1:200) or NG2 (abcam, ab129051; dilution, 1:200) for 20 min at 4 °C. Afterwards, probes were spun down (500× *g*, 7 min), the supernatant was removed, and the pellet suspended in PBS containing Alexa Fluor™ 555-labeled secondary antibodies (Invitrogen, ThermoFisher Scientific, Waltham, MA, USA; goat anti-rabbit; A32733) for 20 min at 4 °C in the dark. Antibodies were removed by centrifugation (500× *g*, 7 min), and cells were resuspended in fixation/permeabilization buffer (eBioscience™, Cat. No. 00-5523-00). Intracellular staining was performed including antibodies against smooth muscle alpha actin conjugated with Alexa Fluor™ 488 (Novus Biologicals via BioTechne GmbH, D-65205 Wiesbaden; NBP2-34760AF488) or against Ki-67 conjugated with Alexa Fluor™ 647 (Invitrogen, ThermoFisher Scientific, Waltham, MA, USA, 17569882). Probes were spun down (500× *g*, 5 min), resuspended in PBS, and directly analyzed on a flow cytometer (ThermoFisher Scientific, Waltham, MA, USA; Attune NxT acoustic focusing cytometer). Data were analyzed for the number of positive cells using FlowJo v10 software and are shown as% of total cells or as the mean fluorescence intensity per cell.

### 2.9. RNA Isolation and Quantitative Real-Time Polymerase Chain Reaction

After cultivation for 6 days, cells were washed once with PBS, and then Trizol reagent (TRI Reagent™ Solution, Ambion via Invitrogen, Austin, TX, USA; Cat. No. AM9738) was added directly to the culture dish (300 µL). Cells were mixed and scraped off the plate using a sterile cell scraper (Greiner via Sigma-Aldrich, Cat. No. 541070) and transferred to 1.5 mL Eppendorf tubes. To extract the RNA, 60 µL of chloroform (Roth, Cat. No. 7331.1) was added to 300 µL of each sample, gently mixed by inversion, and incubated for 10 min at RT. Then, samples were centrifuged (12,000× *g* for 12 min at 4 °C), and the aqueous layer was collected into a fresh tube. To precipitate the RNA, 150 µL of isopropanol (2-propanol; Roth, Cat. No. 6752.1) was added, mixed gently, and kept at RT for 10 min. After centrifugation (12,000× *g* for 8 min at 4 °C), the supernatant was discarded and the RNA pellet washed in 75% ethanol (AppliChem via Fisher Scientific GmbH, D-58239 Schwerte; Cat. No. A3678) by centrifugation (7500× *g* for 5 min at 4 °C). The RNA pellet was air dried under the hood for 10 min to allow any remaining ethanol to evaporate. Then, the ethanol-free pellet was resuspended in RNAse-free water (20–30 µL depending on the size of the pellet). The amount and quality of the RNA was determined at 260 and 280 nm using a spectrophotometer (Nanodrop; ThermoScientific). Quantitative real-time polymerase chain reaction (qPCR) was performed to examine the gene expression of cellular markers. Complementary DNA (cDNA) was transcribed from RNA using the DNase treatment kit (Sigma-Aldrich, D-82024 Taufkirschen, Germany, Cat. No. AMPD1-1KT) and the reverse transcription kit (Promega, Cat. No. M1701). The sequences of all primer pairs used in this study are shown in Table S1. Ct values were used to evaluate relative gene expression levels normalized to housekeeping gene expression.

### 2.10. Western Blot Analysis

Western blot was performed as described in [18]. Membranes were incubated with primary antibodies against PCNA (Santa Cruz Biotechnology, TX 75220, USACat. No. sc-56; dilution 1:1000), smooth muscle alpha actin (Sigma-Aldrich, D-82024 Taufkirschen, Germany, A2547; dilution, 1:1000 in 5% BSA), platelet-derived growth factor receptor alpha and beta (Abcam, Cambridge, UK; Cat. No. ab32570; dilution, 1:1000 in 5% BSA), or Neural/Glial antigen 2 (Abcam, Cambridge, UK; Cat. No. ab129051; dilution, 1:1000 in 5% BSA).

### 2.11. Quantification of Cell Area, Perimeter, and Diameter

Cell morphology was analyzed using ImageJ software from pictures captured with a Keyence microscope at 60× magnification. The pictures were opened in the software and each single cell was analyzed for morphological parameters, including the cell area, perimeter, and minimal and maximal caliper (Feret’s diameter) using the freehand selection tool.

### 2.12. Analysis of Cell Migration

The scratch wound assay was used to explore the migratory abilities of mural cells cultivated for 6 days in different cell culture media. For this, cells were cultivated on 12-well plates (VWR International GmbH, D-64295 Darmstadt, Germany; Cat. No. 734-2325) and grown until 95–100% confluency. A sterile 100 µL pipette tip was used to induce a scratch wound injury across the cell monolayer. Images of the scratch wound were captured immediately afterwards (to document the starting point for observing cell migration, set at 100%) and five hours later using a brightfield microscope (Motic, model AE31, Motic Europe GmbH, D35578 Wetzlar, Germany). The cellular response to a scratch wound was quantified on 10× magnification images by measuring the cell-free area. Results are expressed as the cell-free area at 5 h relative to the cell-free area at the start of the experiment.

### 2.13. Seahorse Metabolic Flux Analysis

To study the metabolic activities of aortic mural cells cultivated in different cell growth media in real time, the Seahorse XF96e Extracellular Flux analyzer (Agilent Technologies, Santa Clara, CA 95051, USA) was used, as described previously. [19] In brief, mural cells were seeded in gelatin-coated XF96 (V3) polystyrene cell culture plates (Agilent Technologies, Santa Clara, CA 95051, USA; Cat. No. 103794-100) in a different culture medium one day before the metabolic rate assay was performed. After 24 h, the medium was replaced with XF assay medium (XF RPMI with 1 mM HEPES; Agilent Technologies, Santa Clara, CA 95051, USA; 103576-100), supplemented with 1 mM pyruvate (Agilent Technologies, 103578-100), 2 mM L-glutamine (Sigma-Aldrich, D-82024 Taufkirschen, Germany, G7513), and 5 mM glucose (Agilent Technologies, Santa Clara, CA 95051, USA; 103577-100). The cells were incubated at 37 °C in a non-CO_2_ incubator for 45 min. Real-time measurements of the extracellular acidification rate (ECAR) and the oxygen consumption rate (OCR) were performed to determine glycolysis and oxidative phosphorylation, at baseline and in response to mitochondrial modulators, including the ATP synthase inhibitor oligomycin (1.5 µM), the electron transport chain complex inhibitors rotenone and antimycin A (0.5 µM each), and the uncoupling agent FCCP (fluoro-carbonyl cyanide phenylhydrazone; 1 µM) provided with the kit. Cell numbers were determined by staining with 20 µM Hoechst 33342 (ThermoFisher Scientific, Waltham, MA, USA; Cat. No. 62249) and detected using the Cytation 5 multimode reader (Agilent BioTek, Santa Clara, CA 95051, USA) with the DAPI filter cube (377/360 nm excitation and 447/460 nm emission). Results were normalized to the cell number per well (1000 cells). Data analysis was performed using Wave 2.6.3.5 Software (Agilent Technologies, Santa Clara, CA 95051, USA).

### 2.14. Cellular Reactive Oxygen Species Detection

Dihydroethidium dye (DHE; 1 µM, Invitrogen, ThermoFisher Scientific, Waltham, MA, USA, Cat. No. D23107) was used to detect the cellular production of reactive oxygen species (ROS) using 4% PFA-fixed cells grown on glass cover slips and incubated for 30 min at 37 °C in the dark. An inverted fluorescence microscope (Keyence, D-68263 Neu-Isenburg, Germany; model BZ-X810) was used to take pictures of the stained cells. Pictures were analyzed at 60× magnification, with eight pictures per sample and condition, using Image software (ImageJ, version 1.54f).

### 2.15. Statistical Analysis

Data were analyzed for normal distribution using the Shapiro–Wilk test and are presented as mean ± standard error of the mean (SEM) or as median and interquartile range, accordingly. If a normal distribution was present, findings in the three experimental groups were compared using one-way ANOVA, Šídák multiple comparisons test. If a normal distribution was not present, Kruskall–Wallis, Dunn’s multiple comparisons test, was used. Two-way ANOVA was used to compare data in more than two groups and an additional variable. A *p* value of less than 0.05 was considered statistically significant. All statistical analyses were performed using GraphPad Prism software (GraphPad Software, Inc.; version 10.1.2; Boston, MA 02110, USA).

## 3. Results

### 3.1. Medium Composition Does Not Affect the Viability of Primary Aortic Mural Cells

**Figure 1:**
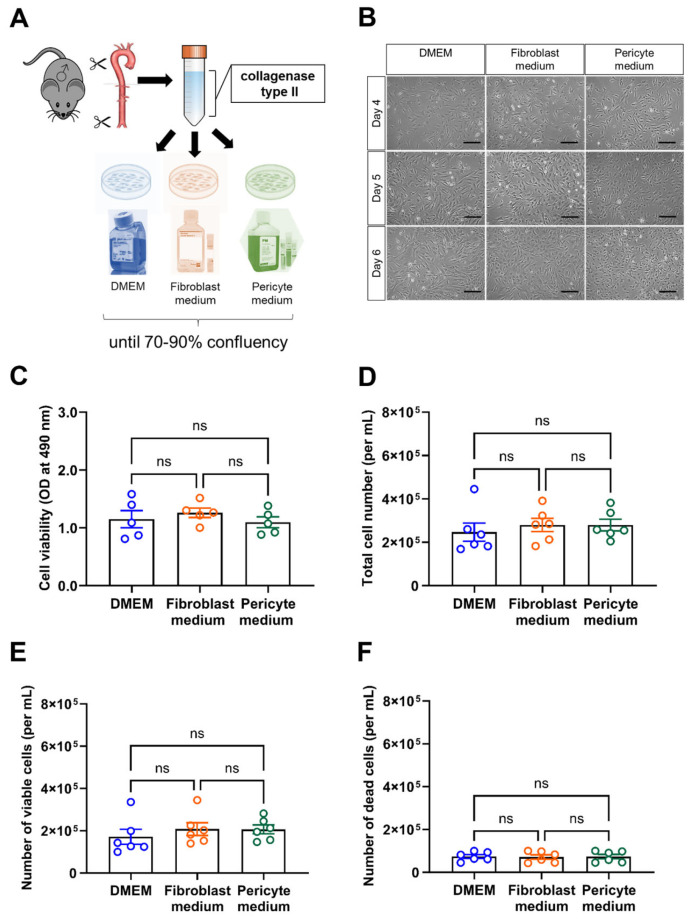
Experimental workflow and assessment of cell viability. (**A**), Schematic drawing of the experimental workflow. Of note, aortic mural cells at passage 0 and 1 (for MTS assay, Seahorse metabolic measurements) were examined in parallel in all experiments. (**B**), Representative brightfield microscopic pictures of mural aortic cells cultivated in different culture media for 4, 5, or 6 days. Scale bars, 10 µm. (**C**), Results of the MTS assay to assess cell viability. Data shown represent the optical density (OD) at 490 nm. (**D**–**F**), the number of total (**D**), viable (**E**) and dead (**F**) cells per mL was automatically determined using the Tecan Spark multimode reader. Data shown represent five (**C**) or six (**D**–**F**) biological replicates per condition. Shapiro–Wilk test was used to test for normal distribution. Statistical significance was assumed if *p* reached a value < 0.05. ns, non-significant (One-way ANOVA, Sidak’s multiple comparisons test (**C**,**E**,**F**), Kruskal–Wallis, Dunn’s multiple comparisons test) (**D**).

Vascular mural cells were isolated from the aorta (including the thoracic and abdominal aorta but excluding the arch) of male C57BL6/J mice and divided into three equal portions to be cultivated in one of three different types of culture medium commonly used for the cultivation of vascular mural cells, that is, DMEM containing 10% FBS, Fibroblast medium, or Pericyte medium. A graphical overview of the experimental setup is shown in Figure 1A. Representative brightfield images of cells cultivated for either 4, 5, or 6 days (without any passaging) in these different media are shown in Figure 1B. After cultivation for 6 days (with medium changes every two days and without passaging), cell viability was examined using the MTS assay. These analyses revealed that the viability of aortic mural cells did not significantly differ between the experimental groups (Figure 1C). Similar findings were obtained using automated quantitative analysis of the total cell number (Figure 1D), and the number of viable (Figure 1E) or dead (Figure 1F) cells using the Tecan Spark multimode microplate reader.

### 3.2. Primary Aortic Mural Cells Expanded in Pericyte Medium Exhibit Increased Proliferation

**Figure 2:**
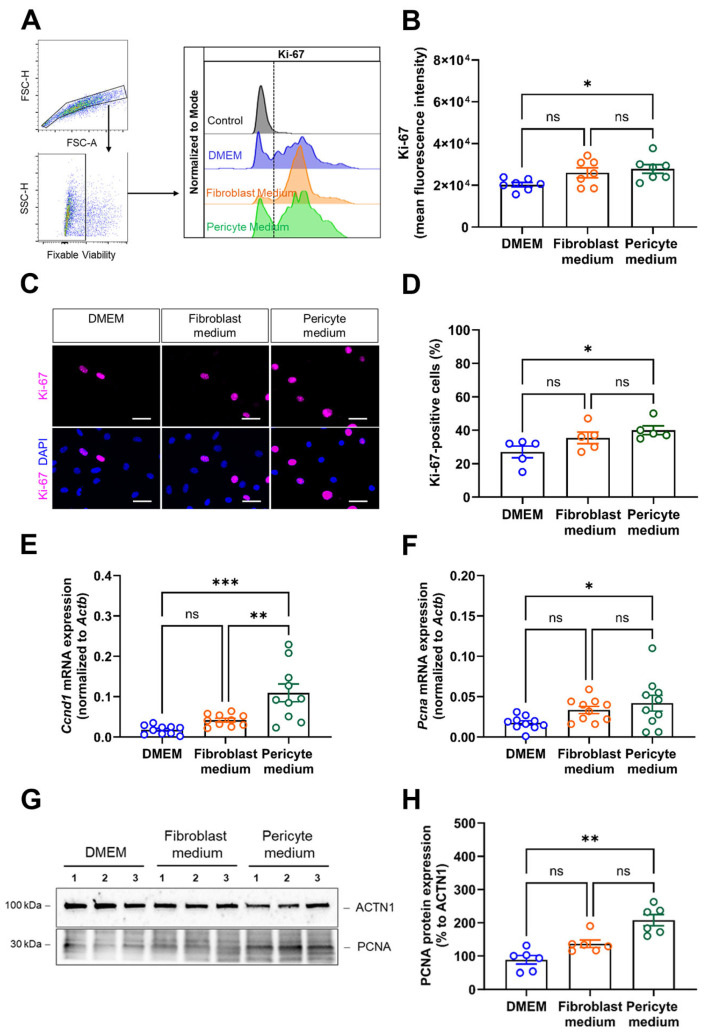
Analysis of cell proliferation. (**A**,**B**), Flow cytometry analysis of Ki-67 was performed to quantify proliferating cells, the gating strategy to detect singlet cells and viable cells as well as representative histogram plots are shown in (**A**), the summary of findings in seven biological replicates in (**B**). (**C**,**D**), Immunostaining of Ki-67 was performed to visualize proliferating cells. Representative pictures are shown in (**C**), the quantitative analysis in (**D**). Data shown represent the mean of eight images (at 60× magnification) collected per sample (five biological replicates), per condition, and are expressed as percentage of total cells. Scale bars, 50 µm. (**E**,**F**), Quantitative real-time PCR analysis of proliferation marker genes. The results after qPCR analysis of *Ccnd1* (**E**) and *Pcna* (**F**) mRNA transcript levels are shown. Data shown represent 10 biological replicates. (**G**,**H**), Immunoblot analysis of PCNA protein expression in aortic mural cells cultivated in DMEM, Fibroblast medium or Pericyte medium for 6 days. Representative findings are shown in (**G**), the results of the quantitative analysis for PCNA in (**H**). Note that the representative images for ACTN1 shown in panel (**G**) are from the same membrane and also shown in Figure 4, panel (**H**) (the uncropped membranes are provided in the supplement). * *p* < 0.05, ** *p* < 0.01, and *** *p* < 0.001 (One-way ANOVA, Sidak’s multiple comparisons test (**B**,**D**–**F**), Kruskal–Wallis, Dunn’s multiple comparisons in (**H**)). ns, non-significant.

Next, we quantified cell proliferation using Ki-67 as a marker. Flow cytometry analysis showed that the mean fluorescence intensity (MFI) of Ki-67 was significantly higher in cells expanded in Pericyte medium (Figure 2A,B). Immunocytochemistry confirmed these findings by showing significantly increased numbers of Ki-67 immunopositive cells cultivated in Pericyte medium compared to DMEM and non-significant changes in cells cultivated in Fibroblast medium (Figure 2C,D). Quantitative PCR analysis of the mRNA levels of cyclin D1 (Ccnd1; Figure 2E) and proliferating cell nuclear antigen (Pcna; Figure 2F), and western blot detection of PCNA (Figure 2G,H) supported findings of increased proliferation in primary aortic cells cultivated under pericyte conditions.

### 3.3. Cultivation of Primary Aortic Mural Cells in Pericyte Medium Is Associated with Telomere Shortening, but Does Not Affect Markers of Apoptosis or Senescence

**Figure 3:**
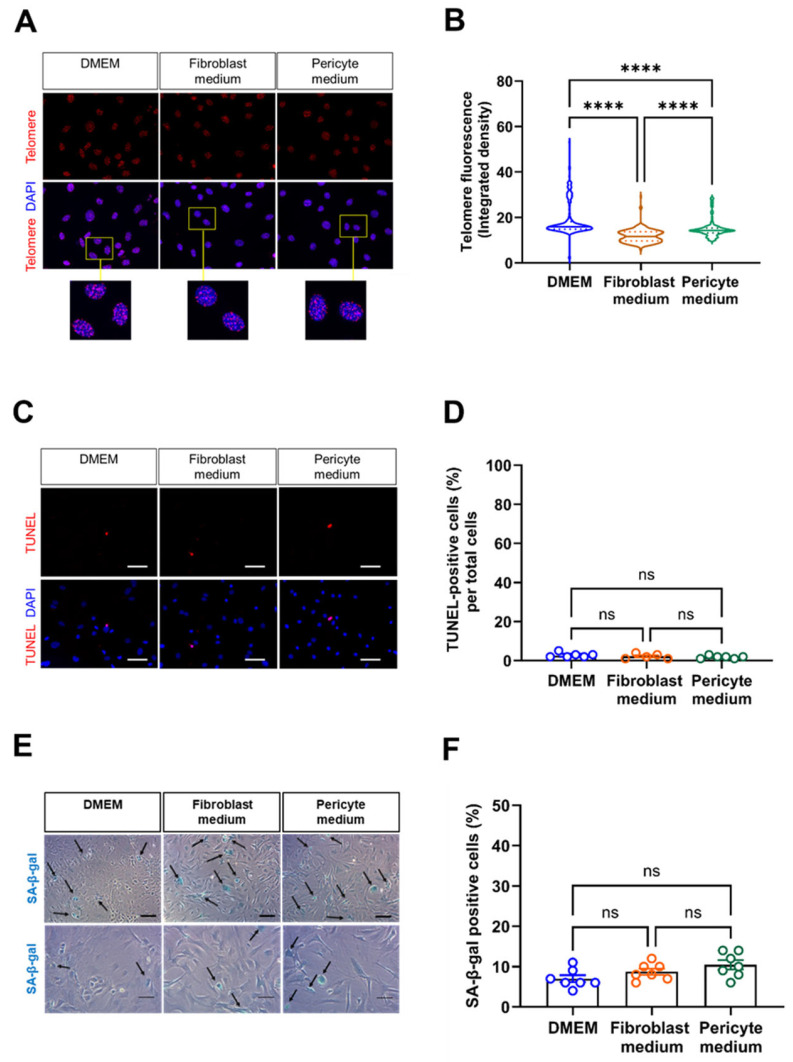
Detection of biologically aged, apoptotic, and senescent cells. (**A**,**B**), Fluorescence in situ hybridization (FISH) was performed to detect the telomere repeat sequence. Representative images are shown in (**A**), the results of the quantitative analysis of the telomere fluorescent intensity in (**B**). Data shown represent two biological replicates, with 220 cell nuclei examined (at 60× magnification) per sample, per condition. Scale bars, 50 µm. (**C**,**D**), Detection of apoptosis-related DNA fragmentation using the TUNEL (terminal deoxynucleotidyl transferase dUTP nick end labeling). Representative fluorescence microscopy images (**C**) and the results of the quantitative analysis (**D**). Scale bars, 20 µm. Data shown represent six biological replicates, with five images (at 20× magnification) collected per sample, per condition, and are expressed as percentage of total cells. (**E**,**F**), Senescence-associated beta-galactosidase (SA-β-gal) staining was used to assess the presence of senescent cells (arrows). Representative images of two different samples and magnifications are shown in (**E**), the results of the quantitative analysis in (**F**). Scale bars, 10 µm (top row) and 20 µm (bottom row). Data shown represent seven biological replicates per condition, with one image (at 10× magnification) collected per sample, per condition. **** *p* < 0.0001 (Kruskal–Wallis, Dunn’s multiple comparisons test in (**B**,**D**), One-way ANOVA, Sidak’s multiple comparisons test in (**F**)). ns, non-significant.

Next, we examined indicators of biological aging. Fluorescence in situ hybridization (FISH) revealed a significantly reduced telomere length in cells expanded in Fibroblast and Pericyte medium compared to those cultivated in DMEM (Figure 3A,B). On the other hand, the number of apoptotic cells, visualized by fluorescence microscopy analysis of TUNEL-positive cells, was low and similar in cells cultivated under different media conditions (Figure 3C,D). Messenger RNA levels of Tp53 (encoding for tumor suppressor protein 53) also did not significantly differ between the groups. The number of SA-β-gal-positive cells was low and similar at this time point, that is at passage 0 (Figure 3E,F), suggesting that none of the culture media induced cellular senescence.

### 3.4. The Expression of Marker Genes for Differentiated Smooth Muscle Cells Is Best Preserved in Primary Aortic Mural Cells Expanded in Basal Medium

**Figure 4:**
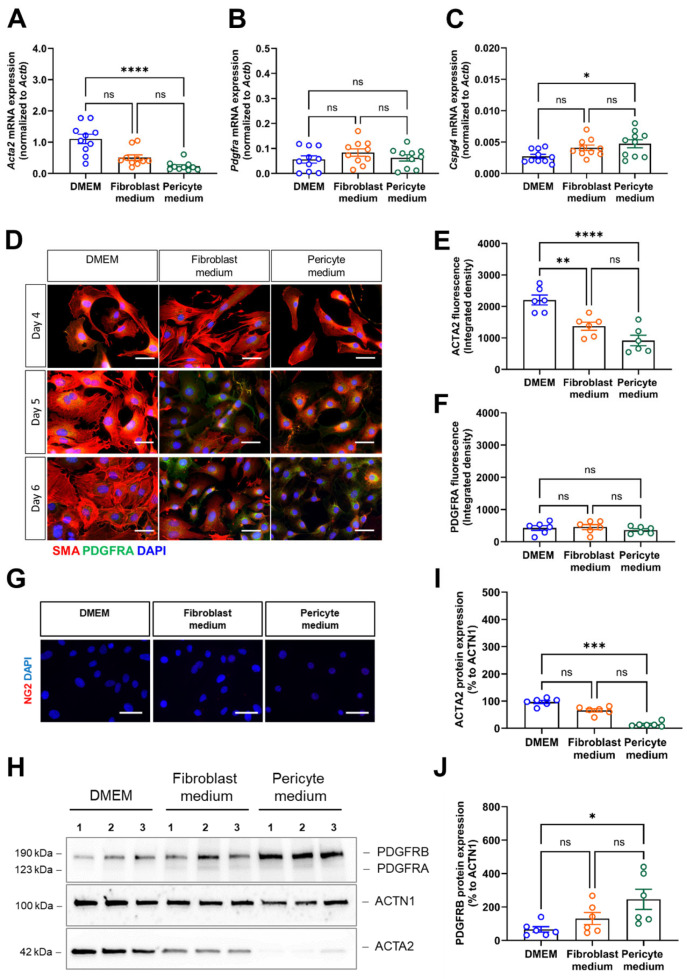
Expression of vascular mural cell lineage markers. (**A**–**C**), Quantitative real-time PCR analysis was used to determine the mRNA transcript levels of the smooth muscle cell marker *Acta2* (**A**), the fibroblast marker *Pdgfra* (**B**) and the pericyte marker *Cspg4* (**C**). Data shown represent ten biological replicates. (**D**–**F**), Immunofluorescence staining of SMA (red fluorescence) and PDGFRA (green fluorescence). Representative images (**D**) and quantitative analysis of SMA in (**E**) and PDGFRA in (**F**). Data shown represent the integrated fluorescence density in six biological replicates, with mean of eight images (at 60× magnification) examined per sample, per condition. Scale bars, 50 µm. (**G**), Representative images after immunofluorescence staining of NG2 (red fluorescence). (**H**–**J**), Immunoblot analysis of vascular mural markers. A representative membrane (**H**) and the results of the quantitative immunoblot analysis of ACTA2 (**I**) and PDGFRB (**J**) protein expression in five biological replicates per condition are shown. Note that the representative images for ACTN1 shown in panel (**H**) are from the same membrane and also shown in Figure 2, panel (**G**) (the uncropped membranes are provided in the supplement). * *p* < 0.05, ** *p* < 0.01, *** *p* < 0.001 and **** *p* < 0.0001 (One-way ANOVA, Kruskal–Wallis, Dunn’s multiple comparisons test in (**A**,**I**); Sidak’s multiple comparisons test in (**B**,**C**,**E**,**F**,**J**)). ns, non-significant.

We then also examined the expression of marker genes commonly used to distinguish smooth muscle cells (i.e., actin alpha 2, smooth muscle, Acta2), fibroblasts (i.e., platelet-derived growth factor alpha, Pdgfra), and pericytes (i.e., chondroitin sulfate proteoglycan 4, Cspg4, also known as Ng2), respectively. Quantitative real-time PCR analysis showed that Acta2 mRNA levels were highest in cells cultivated in DMEM compared to those cultivated in Pericyte medium (Figure 4A). Pdgfra mRNA expression levels were low and did not significantly differ between the different culture media (Figure 4B), whereas Cspg4 mRNA transcripts significantly increased in cells cultivated in Pericyte medium compared to DMEM, albeit at very low levels (Figure 4C). Immunostaining of cells cultivated under different medium conditions for 4,5, and 6 days confirmed high SMA protein expression in primary aortic mural cells cultivated in DMEM, while SMA immunosignals decreased from day 4 to day 6 in cells expanded in Fibroblast or Pericyte medium (representative findings are shown in Figure 4D, the results of the quantitative analysis in Figure 4E). In line with the mRNA data, cellular PDGFRA immunosignals were low (Figure 4D,F), and NG2 immunosignals were largely undetectable and therefore not quantified (Figure 4G). Western blot analysis confirmed these findings (Figure 4H) and showed significantly lower SMA protein levels in cells expanded in Pericyte medium compared to DMEM (Figure 4I), whereas protein levels of PDGFRA were low in all conditions (Figure 4H) and NG2 was not detected. Of note, protein levels of PDGFRB, expressed on fibroblasts and pericytes and detected by the same antibody, significantly increased in cells cultivated in Pericyte medium (Figure 4J).

**Figure 5:**
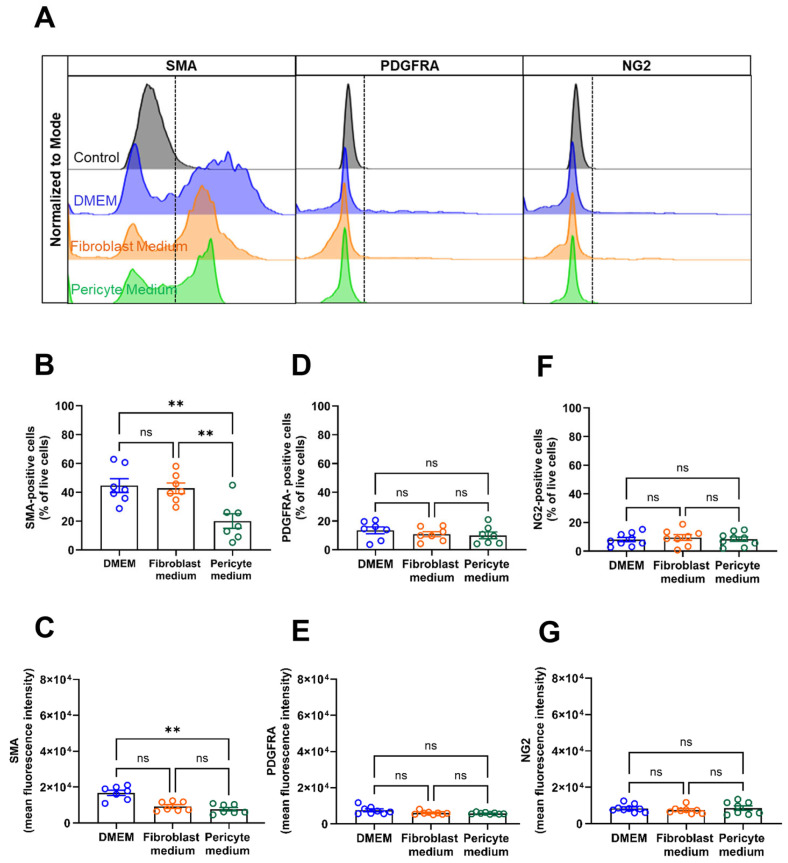
FACS analysis of vascular mural markers. (**A**–**G**), Flow cytometry analyses was used to study the percentage of immunopositive cells (per total, viable cells) and the mean fluorescence intensity (MFI) of vascular mural markers in cells cultivated cells for 6 days under different media conditions. Representative histogram plots of cells stained for smooth muscle alpha actin (SMA), platelet-derived growth receptor alpha (PDGFRA), or neural/glial antigen 2 (NG2) are shown in (**A**). The percentage and MFI after staining for SMA is shown in (**B**,**C**), for PDGFRA in (**D**,**E**), and for NGs in (**F**,**G**). Data shown represent seven biological replicates per condition. ** *p* < 0.01 (One-way ANOVA, Sidak’s multiple comparisons test in (**B**,**D**–**G**); Kruskal–Wallis, Dunn’s multiple comparisons test in (**C**)). ns, non-significant.

Flow cytometry analysis representative histogram plots are displayed in Figure 5A confirmed the above findings by showing significantly higher numbers of SMA-immunopositive cells in primary aortic mural cells cultured in DMEM, both expressed as the percentage of total cells (Figure 5B) and as MFI (Figure 5C), whereas expression levels of PDGFRA (Figure 5A,D,E) and NG2 (Figure 5A,F,G) were low and similar in cells grown under the different culture conditions.

**Figure 6:**
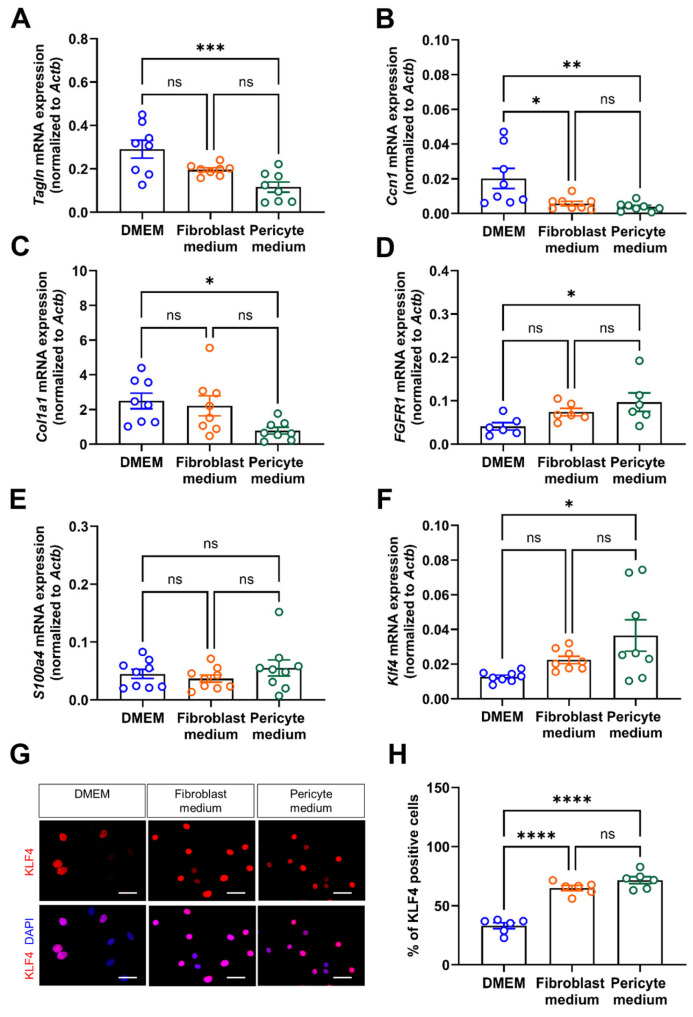
mRNA expression of vascular mural differentiation markers. (**A**–**F**), Quantitative real-time PCR analysis to determine the mRNA transcript levels of transgelin (*Tagln*; (**A**)), calponin (*Cnn1*; (**B**)), collagen 1A type 1 (*Col1a1*; (**C**)), fibroblast growth factor receptor 1 (*Fgfr1*; (**D**)), S100 calcium binding protein A4 (*S100a4*; (**E**)), and Krüppel-like factor 4 (*Klf4*; (**F**)). Data shown represent findings in six (**D**) or eight (**A**–**C**,**E**,**F**) biological replicates. (**G**,**H**), Immunofluorescence staining of nuclear KLF4. Representative images (**G**) and quantitative analysis (**H**). Data shown represent six biological replicates, with eight images (at 60× magnification) examined per sample, per condition. Scale bars, 50 µm. * *p* < 0.05, ** *p* < 0.01, *** *p* < 0.001 and **** *p* < 0.0001 (One-way ANOVA, Sidak’s multiple comparisons test). ns, non-significant.

Quantitative PCR analysis of additional markers indicative of SMC differentiation, such as transgelin (*Tagln*; Figure 6A), or involved in the negative regulation of vascular smooth muscle cell proliferation, such as calponin-1 (*Cnn1*; Figure 6B), or of collagen type 1 A1 (*Col1A1*; Figure 6C), also showed significantly higher mRNA levels in primary aortic mural cells cultivated in DMEM compared to those cultivated in Pericyte medium. On the other hand, mRNA levels of *Fgfr1* were significantly increased in cells expanded in Pericyte medium (Figure 6D). Markers of inflammatory fibroblast activation, such as fibroblast specific protein 1 (S100 calcium binding protein a4, *S100a4*; also known as *Fsp1*) [20] did not significantly differ between the experimental groups (Figure 6E). Findings of increased mRNA levels of Krüppel-like factor 4 (*Klf4*) (Figure 6F) and increased nuclear immunosignals of KLF4 (Figure 6G,H) in cells cultivated in Pericyte medium supported the observed loss of differentiation markers under these conditions.

### 3.5. Primary Aortic Mural Cells Expanded in Pericyte Medium Are Smaller and Exhibit an Increased Migratory Capacity

**Figure 7:**
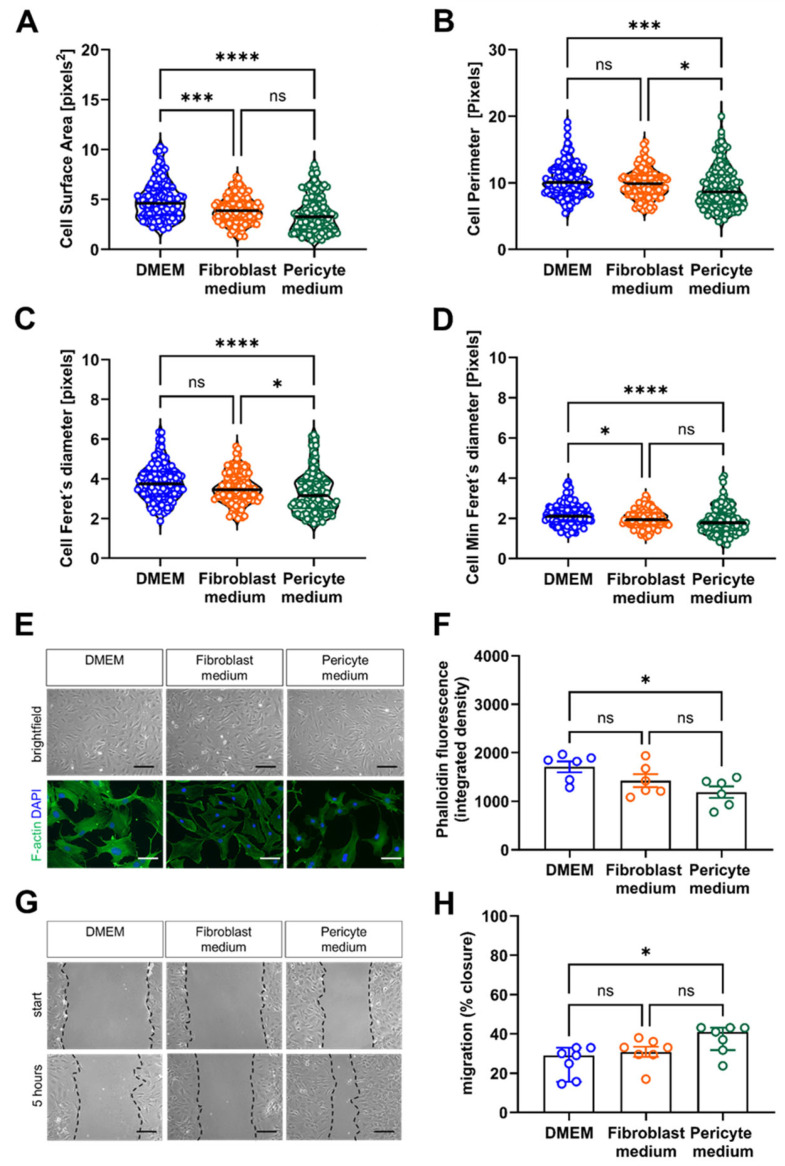
Analysis of cytoskeletal remodeling and cell migration. (**A**–**D**), Automated quantitative image analysis of cell size and shape. The results of the ImageJ based automated quantification of the single cell area (**A**), perimeter (**B**), Feret’s diameter (**C**) and min Feret’s diameter (**D**) are shown. Data shown represent three biological replicates, with six images (60× magnification) per sample, per condition. (**E**,**F**), Analysis of cytoskeletal rearrangements associated with cell motility. Representative brightfield images of unstained cells (top row) and after staining with fluorescein phalloidin (bottom) (**E**) and the results of the quantitative analysis (**F**). Data shown represent six biological replicates, with eight images (60× magnification) per sample, per condition. (**G**,**H**), Analysis of cell migration using the scratch wound assay. Representative images immediately after inducing a ‘scratch wound’ on monolayer cells (start) and 5 h later (**G**). Black, interrupted lines were added to indicate the cell borders. Quantification of the cell-free area at five hours relative to the area at start (**H**). Data shown represent seven biological replicates, with two images (10× magnification) per sample, per condition. Scale bars: 50 µm. * *p* < 0.05, *** *p* < 0.001 and **** *p* < 0.0001 (Kruskal–Wallis, Dunn’s multiple comparisons test in (**A**–**D**); One-way ANOVA, Sidak’s multiple comparisons test in (**F**,**H**)). ns, non-significant.

Cytoskeletal re-organization and the migration of cells from the media and outer layers of the vessel wall is an important event during atherosclerosis or restenosis lesion formation [21]. Quantitative image analysis of the effects of the culture medium on the morphology (size and shape) of single cells revealed that the surface area (Figure 7A), perimeter (Figure 7B) as well as the longest (Figure 7C) and shortest (Figure 7D) distance between two points were largest in cells cultivated in DMEM compared to Fibroblast and Pericyte medium, in line with an increase in total cell size but not shape. Staining of F-actin fibers using fluorescein-labeled phalloidin to visualize the cytoskeleton showed the lowest signals in cells grown in Pericyte medium (Figure 7E,F). Quantitative analysis of the relative closure five hours after the induction of a scratch wound revealed that primary aortic mural cells cultivated in Pericyte medium exhibited a significantly faster wound closure than cells grown in Fibroblast medium or DMEM (Figure 7G,H). Collectively, these data suggested that cultivation in Pericyte medium enhances the migratory capacities of primary mural aortic cells.

### 3.6. Expansion of Primary Aortic Mural Cells in Fibroblast Medium Is Associated with Metabolic Reprogramming

**Figure 8:**
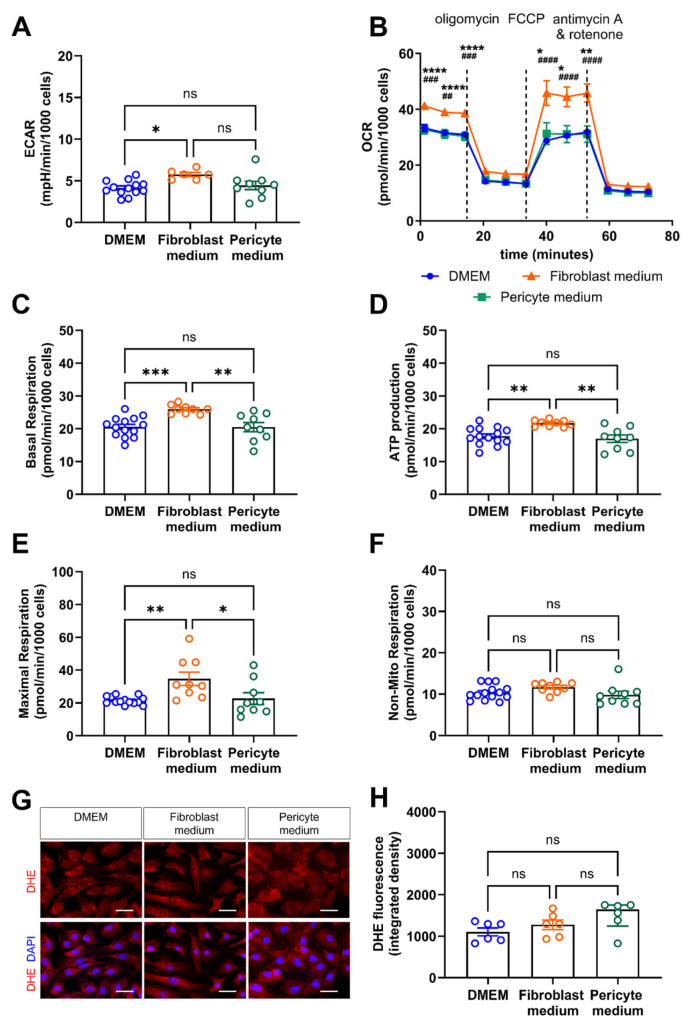
Real-time assessment of cell metabolism and oxidative species generation. (**A**–**F**), Cellular bioenergetics were studied using the Seahorse Real-Time Cell Metabolic Analyzer. (**A**) Extracellular acidification rate (ECAR) as a measure of glycolysis. (**B**) Oxygen consumption rate (OCR) to determine oxidative phosphorylation, alone and in response to metabolic stress induced by oligomycin, FCCP (fluoro-carbonyl cyanide phenylhydrazone), and antimycin A/rotenone. Data shown are representative of results obtained in three biological replicates. * *p* < 0.05, ** *p* < 0.01, *** *p* < 0.001 and **** *p* < 0.0001 for Fibroblast medium vs. DMEM, ## *p* < 0.01, ### *p* < 0.001 and #### *p* < 0.0001 for Fibroblast medium vs. Pericyte medium (Two-way ANOVA, Tukey’s multiple comparisons test B). ns, non-significant. Results after quantification of the basal respiration (**C**), ATP production (**D**), maximal respiration (**E**), and non-mitochondrial respiration (**F**) are shown. (**G**,**H**), Dihydroethidium (DHE) immunofluorescence staining to visualize the formation of reactive oxygen species (ROS). Representative images in (**G**), results after quantification of the integrated intensity using ImageJ in (**H**). Data shown represent six biological replicates, with 6 images (at 60× magnification per sample, per condition). Scale bars, 50 µm. * *p* < 0.05, ** *p* < 0.01, *** *p* < 0.001 and **** *p* < 0.0001 One-way ANOVA, Sidak’s multiple comparisons test in (**A**,**C**–**E**); Kruskal–Wallis, Dunn’s multiple comparisons test in (**F**,**H**).

Finally, we examined how the cultivation of primary aortic mural cells under different growth factor conditions affects their metabolism, that is, glycolysis and oxidative phosphorylation, as a source of energy substrates and biosynthetic precursors necessary during cell division and migration. Seahorse metabolic analyzer measurements of the extracellular acidification rate (ECAR; Figure 8A) and the oxygen consumption rate (OCR; Figure 8B) revealed that cells expanded in Fibroblast medium exhibited a significantly increased basal glycolytic activity and oxidative phosphorylation compared to cells expanded in DMEM or in Pericyte medium. Injection of oligomycin uncovered that the elevated basal respiration observed in cells expanded in Fibroblast medium (Figure 8C) was due to increased mitochondrial ATP production (Figure 8D). The maximal respiration also was significantly increased in cells cultured in Fibroblast medium, as detected by injection of the uncoupling agent FCCP (carbonyl cyanide-4 (trifluoromethoxy) phenylhydrazone) (Figure 8E). Non-mitochondrial respiration did not differ between cells grown in different culture medium (Figure 8F). In line, dihydroethidium (DHE) staining showed similar levels of reactive oxygen species production in cells expanded in the three different culture media (Figure 8G,H).

## 4. Discussion

In this study, we show the that cultivation of primary murine aortic mural cells for up to one week in cell culture media with different compositions has significant effects on their proliferation rate, migratory capacity, telomere length, energy metabolism, and the expression of markers of vascular mural cell lineage or differentiation, whereas cellular viability and markers of cellular apoptosis or senescence did not differ at this time point, that is, passage 0. Although our finding that media composition has an impact on the phenotype and specific functions of cultivated cells may seem unsurprising, culture conditions continue to be an important experimental variable that is frequently overlooked. Our data also suggest that cultivating primary aortic mural cells in media containing different growth factors can both provoke and hide phenotypic or functional changes and thus be used to examine cellular plasticity and the response to external stimuli, such as those observed in disease models or in patients.

Cellular differentiation and de-differentiation are central events in the stromal-vascular damage response. According to the response to injury theory first published in the mid-1970s [4,5], an ‘injurious agent’ initiates a series of cellular and molecular events leading to atherosclerosis and other vascular disease processes. PDGF released from activated platelets was identified as central factor and potent mitogen stimulating the proliferation of SMCs in vivo and in culture [22]. PDGFRB signaling is successfully targeted to treat patients with proliferative malignancies originating from hematopoietic and stromal vascular cells [23]. To this day, primary cells in culture are still being used to study the molecular mechanisms of the vascular response to injury or signaling pathways in vascular mural cells during phenotype switching. Expanding primary cells in culture media differing in the types and amounts of growth factors will therefore not only drive them toward a certain state of differentiation, as intended or maybe not, but also affect their phenotype and function examined in different experimental readouts.

The development of atherosclerotic or restenotic vascular lesions involves the transition of vascular SMCs from a quiescent state to an activated phenotype, a central mechanism underlying their contribution to vascular disease [24]. Platelets are an important source of growth factors, and their release from platelet storage granules following activation plays an important role in modulating the differentiation and proliferation of vascular cells [25]. In addition to PDGF, other potent growth factors released from activated platelets include FGF, VEGF, and TGFβ. They are, at different concentrations and combinations, contained as recombinant proteins in complete medium preparations used for vascular mural cell cultivation and expansion. PDGF is a powerful mitogen, but it also induces SMC de-differentiation and migration [26]. In addition to being stored in platelets, vascular SMCs themselves also express PDGF, primary the A chain, whereas only minute amounts of mRNA transcripts for the PDGF-B chain have been detected [27]. FGF also increases the proliferation and migration of SMCs and induces a phenotype change from a contractile to a synthetic state [28]. Other platelet-derived factors with potential effects on vascular mural cells, such as CD40L [29,30] or CCL5 (RANTES) [31] and CXCL4 (PF4) [32] are not routinely added to commercial cell culture medium, as they may direct differentiation toward more proinflammatory (CD40L) or atherogenic (CCL5) endotypes.

Vascular mural cells also express and secrete growth factors such as VEGF, which primarily act on endothelial cells [33]; however, VEGF receptors are also expressed on vascular mural cells [33]. For example, VEGF-B binds and signals via FGF type 1 receptors (FGFR1) [34], and a large number of studies has documented VEGF activities on both fibroblasts (over 9500 citations in PubMed, as of May 2025) and pericytes [35]. In the present study, we observed increased FGFR1 mRNA levels in cells cultivated in Pericyte medium. Unfortunately, the composition of the Pericyte medium supplement was not disclosed by the company. Access to such information should be considered as a criterion when selecting the experimental tools.

Serum is another important component of cell growth media, although the cultivation of SMCs is possible in the complete absence of added serum [16]. In fact, the stepwise reduction of serum concentrations may be used to induce SMC de-differentiation [21] A previous study comparing different medium conditions for the cultivation of vascular mural stromal cells found that ‘while all techniques supported the establishment of cultures to varying degrees, sustained growth and serial propagation were only achieved in 10% FBS medium’ [36], and our data corroborate these previous findings. In our study, changes in the expression of SMA and other markers of SMC differentiation, such as transgelin or calponin, were mirrored by observations of altered proliferation, showing the highest degree of quiescence and differentiation in primary mural aortic cells cultivated in DMEM as opposed to an increased proliferation and de-differentiation of cells cultivated in Pericyte medium. Findings of significant reductions in telomere length under these cultivation conditions highlight their potential impact on cellular senescence and aging as well as the factors and cell types driving these processes. The importance of fibroblast or SMC senescence for myofibroblast transdifferentiation [37] and vascular disease of the kidney [38] or the lung [39,40] has already been shown. Metabolic activities were enhanced, particularly in cells cultivated in Fibroblast growth medium, supporting a mechanistic link between proliferation, metabolism, and metabolic aging and its impact on vascular disease [41], whereas the discrepant findings regarding proliferation/telomere shortening and metabolic activities in cells cultivated in Pericyte medium suggest additional mechanisms. The effect of cell density on the synthesis of SMC phenotype markers in primary cultures was described earlier [42]. Faster doubling rates in cells cultured in Pericyte medium have been reported by others [43]. The significantly upregulated expression of FGFR1, shown to promote cell cycle progression in human vascular mural cells [44], also may have played a role. Expression levels and nuclear immunosignals of KLF4, a transcriptional repressor of genes involved in differentiation in vascular mural cells [45], also were most strongly upregulated in cells expanded in Pericyte medium. KLF4 upregulation in combination with the loss of marker expression, increased proliferation, migration, and ECM synthesis, as seen in our study in cells expanded in Pericyte medium, was reported in perivascular cells in response to tumor-secreted factors [46]. The observed increase in mRNA levels of *Col1A1*, the main collagen type in vascular fibrosis [3], in aortic mural cells cultured in DMEM is also remarkable and potentially relevant for studies examining fibrosis. Of note, expression levels of the pericyte marker NG2 were near to undetectable in our study, and the expression of PDGFRA, a marker of fibroblasts, also was low and similar among cells expanded in different cell media. On the other hand, we observed strong expression of PDGFRB, expressed on pericytes as well as fibroblasts, and protein expression levels were highest in cells cultivated in Pericyte medium. Although the appearance of pericytes in culture may take longer than six days, there are no pericyte-specific markers and they also vary between different organs [47].

Cells of diverse origins are known to contribute to fibrosis [48]. Fibroblast is a common term for cells originating from different cellular sources and biological processes, including the activation of resident fibroblasts, differentiation of hematopoietic or vascular mural stem cells, but also the de-differentiation of SMCs, pericytes or adipocytes, or the transdifferentiation of endothelial cells toward the vascular mural cell lineage, among others. Signals promoting fibroblast activation include profibrotic cytokines and growth factors (such as angiotensin II, endothelin-1, or tumor necrosis factor-α), which can be added, if not already present, to the culture media. The profibrotic effects of mechanical forces [49] or hypoxia [50] can also be modeled in vitro, but require specific equipment. Interestingly, cultivation of cells in Fibroblast medium conditions increased their glucose metabolism and ATP generation, the latter also under conditions of mitochondrial stress. The supplementation of media with anabolic growth factors, such as insulin, may have contributed to this finding, but differences between cell types regarding metabolic pathways used for energy generation also may have played a role. Whether the changes in cell metabolism may have contributed to the observed significant reduction in telomere length remains to be shown. Recent studies highlighted the importance of metabolic adaptations of vascular mural cells during differentiation [51] and proliferation [52], and the relevance of changes in cell metabolism for vascular disease processes is emerging [41]. Although we did not observe differences in cellular oxidative stress levels between different media, we have to acknowledge that only one out of several available detection tools was used and that additional readout would have been necessary to provide a definite answer.

## 5. Conclusions

The results of our study underline the plasticity of primary aortic mural cells and their ability to adapt to external stimuli, as present in commercial culture media, and also suggest that cultivation conditions need to be carefully evaluated when interpreting the data. Regarding their impact on future vascular biology research, chemically-defined growth media that more closely resemble the conditions observed in disease models or in patients should be developed.

## Data Availability

Data supporting the findings of this study are available from the corresponding author upon reasonable request.

## Supplementary Materials

The following supporting information can be downloaded at: https://www.mdpi.com/article/10.3390/cells14120927/s1: Table S1: Oligonucleotide primer sequences for murine genes.

## Abbreviations

The following abbreviations are used in this manuscript:

DAPI	4′,6-diamidino-2-phenylindole
DHE	Dihydroethidium
DMEM	Dulbecco’s Modified Eagle Medium
ECAR	extracellular acidification rate
ECM	extracellular matrix
FBS	fetal bovine serum
FCS	fetal calf serum
FGF	fibroblast growth factor
FISH	fluorescence in situ hybridization
MFI	mean fluorescent intensity
OCR	oxygen consumption rate
PDGF	platelet-derived growth factor
PFA	paraformaldehyde
qPCR	quantitative real-time polymerase chain reaction
RT	room temperature
SMC	smooth muscle cell
TUNEL	dUTP nick-end labeling
