# Supplementary material for: Side-by-Side Comparison of Culture Media Uncovers Phenotypic and Functional Differences in Primary Mouse Aortic Mural Cells
Source: Cells. 2025 Jun 19;14(12):927. doi: 10.3390/cells14120927 (PMC12190375; doi:10.3390/cells14120927)
Supplement: Supplementary file 1 [file cells-14-00927-s001.zip › cells-3675544-supplementary.pdf]

Table S1. Oligonucleotide primer sequences for murine genes

Gene symbol	Forward primer (5' - 3' direction)	Reverse primer (5' - 3' direction)
Acta2	GGACGTACAACTGGTATTGTGC	CGGCAGTAGTCACGAAGGAAT
Ccnd1	GTTCGTGGCCTCTAAGATGAAGGA	CACTTGAGCTTGTTCACCAGAAGC
Cnn1	CCAGCATGGCCAAGACAA AAG	GGATCATAGAGGTGACGCCG
Col1a1	ATGGATTCCCGTTCGAGTACG	TCAGCTGGATAGCGACATCG
Cspg4	AGGACCTAACATTCCGGGTCA	CTGTGTTGTGGAGGATCTGTATG
Fgfr1	TGTTTGACCGGATCTACACACA	CTCCCACAAGAGCACTCCAA
Klf4	GAAATTCGCCCGCTCCGATGA	CTGTGTGTTTGCGGTAGTGCC
Pdgfra	TATCCTCCCAAACGAGAATGAGA	GTGGTTGTAGTAGCAAGTGTACC
Pdgfrb	ACGGCTGAGCTGAGTGATTC	CATCTCCCAGTGTCTCCAG
S100a4	CTGGGGAAAAGGACAGATGA	TGCAGGACAGGAAGACACAG
Tagln	GACTGACATGTTCCAGACTGTTGAC	CAAACTGCCCAAAGCCATTAG
